# Simulation and Modeling Thrombotic Occlusion in Peripherally Inserted Central Catheters

**DOI:** 10.1111/anec.70090

**Published:** 2025-05-15

**Authors:** Feng‐Xian Li, Qiao‐hong Guo

**Affiliations:** ^1^ College of Nursing Capital Medical University Beijing China; ^2^ Department of Oncology, Beijing Shijingshan Hospital Teaching Hospital of Capital Medical University Beijing China

**Keywords:** model, peripherally inserted central catheter, PICC occlusion

## Abstract

**Objective:**

To simulate thrombotic occlusion of catheters and develop a model for thrombotic occlusion in peripherally inserted central catheters (PICC), providing a framework for research on catheter occlusion and post‐occlusion recanalization.

**Methods:**

Following preparatory steps prior to modeling, sterile anticoagulant bovine blood was drawn and injected into the PICC. Subsequently, the catheter tip was clamped and left to stand for 72 h.

**Results:**

A total of 140 catheter models were produced, all of which exhibited thrombosis, resulting in a 100% success rate for intra‐catheter thrombus production. Of these, 118 models experienced no blood reflux when the syringe plunger was withdrawn and triggered an infusion pump alarm, achieving a catheter occlusion modeling success rate of 84.29%. There were 127 cases where syringe plunger withdrawal resulted in no blood reflux within the thrombotic catheter occlusion models, yielding an incidence rate of 90.71%, while 13 cases revealed blood reflux mixed with fine thrombosis, with an incidence rate of 9.29%. Additionally, 126 models triggered infusion pump alarms, with an incidence rate of 90%, while 14 models did not trigger alarms due to thrombus overflow at the catheter tip, with an incidence rate of 10%. The infusion pump alarm method and the syringe withdrawal method demonstrated a significant correlation in diagnosing thrombotic catheter occlusion.

**Conclusion:**

The method for modeling thrombotic catheter occlusion used in this study is reliable, producing a model that accurately simulates the fundamental characteristics of thrombotic catheter occlusion. This model has the potential for application in clinical practice.

## Graphical abstract

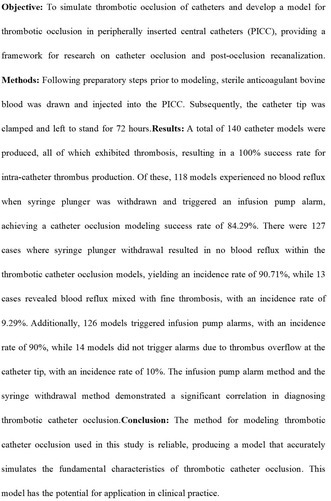

**Abbreviation**
PICC: peripherally inserted central catheters

## 1 Background

Peripherally inserted central catheters (PICC) serve as long‐term infusion devices, particularly valuable in oncology for administering chemotherapy, targeted therapies, and supportive medications like antibiotics and pain management drugs. However, due to their prolonged indwelling time and the complexity and variety of infusion fluids, catheter occlusion occurs frequently. In cancer patients, the risk of occlusion is further heightened by the frequent use of viscous solutions, such as parenteral nutrition and blood products, as well as the potential for drug precipitates. Regular flushing with saline or heparin solutions, along with meticulous catheter care, is essential to maintain patency and prevent complications. Despite these challenges, PICCs remain a crucial tool in oncology, providing reliable venous access for prolonged and complex treatment regimens. Based on the Intravenous Therapy Manual, PICC occlusion is characterized by an inability to withdraw blood, inability to flush or transfuse, slow blood flow, and increased occlusion alarms when using an infusion pump (Gorski 2018). If left untreated, PICC occlusion can lead to the loss of venous access, increased costs associated with catheter replacement, a heightened risk of catheter‐associated infections and thrombosis, and potentially lead to pulmonary embolism. Therefore, addressing PICC occlusion has become an urgent priority.

Baskin revealed that catheter occlusion and catheter‐related thrombosis are the most common complications associated with the use of central venous catheters (Baskin et al. 2009). Catheter occlusion can manifest as either partial blockage, where withdrawal of the syringe plunger results in no blood reflux but infusion is still possible, or complete blockage, where neither blood reflux nor infusion can occur. Catheter occlusion secondary to thrombosis‐like fibrin sheath formation around the catheter tip, intraluminal blood clots, or venous thrombosis can occur independently or in combination. Intraluminal thrombosis accounts for 5%–25% of all catheter occlusions and can lead to complete catheter blockage. Liu has demonstrated that the accumulation of blood clots within the catheter can result in intraluminal thrombus formation (Liu et al. 2023). Hitchcock has identified that the causes of intraluminal occlusion of PICC include blood stagnation within the catheter or blood regurgitation into the PICC lumen, both of which can result in intraluminal thrombosis in the catheter (Hitchcock 2016).

Thrombotic catheter occlusion is identified as the primary cause of catheter occlusion, characterized by a high incidence rate as yet underrepresented in clinical research (Gorski 2018). The study of recanalization procedures for catheter occlusion includes observing thrombus changes during flushing and catheter sealing operations, dynamic thrombosis changes, thrombus overflow, catheter dysfunction, and changes in infusion pressure during occlusion, necessitating direct in vitro observation of the catheter. Therefore, the development of a reliable in vitro model for thrombotic catheter occlusion is crucial. Currently, there is limited research both domestically and internationally concerning such models.

The objective of this study was to establish an in vitro model of thrombotic catheter occlusion to identify the characteristics of catheter occlusion that occur due to thrombosis. This model intends to serve as a reference for effectively preventing and managing thrombotic catheter occlusion in clinical practice.

## 2 Materials and Methods

### 2.1 Experimental Materials

PICCs (BD company): A total of 140 pieces of 4Fr models, constructed from silicone material, with a length of 35 cm.

Infusion joints (BD company): A total of 140 pieces, characterized by balanced pressure and transparency.

1, 5, 10 mL syringes (Wego Jierui).

Tee joint (B. Braun Melsungen AG).

Infusion apparatus (Wego Jierui).

Infusion pump (Beijing KellyMed Co. Ltd.), model (KL‐8052N).

Sterile anticoagulant bovine blood, 100 mL (Zhengzhou Pingrui Biotechnology Co. Ltd.), containing 6 mg of heparin sodium anticoagulant (concentration ≥ 180 usp/mg). The hematocrit was 35%, the erythrocyte sedimentation rate was 7 mm/h, and the pH value was 7.73.

Vitamin K1 injection (10 mg/1 mL) (Jiangsu Huayang Pharmaceutical Co. Ltd.).

Thrombin powder (5000 units/bottle) (Zhejiang Hacon Pharmaceutical Co. Ltd.).

0.9% sodium chloride, 100 mL/bottle (Sichuan Kelun Pharmaceutical Co. Ltd.).

### 2.2 Modeling Method

In this study, the thrombotic catheter occlusion model was produced using PICC and sterile anticoagulant bovine blood. The procedure was as follows:

1. Refrigerated sterile anticoagulant bovine blood was placed in a 37°C water bath and warmed to 37°C.
2. A clean and dry PICC connection infusion joint was prepared.
3. To 3.5 mL of 0.9% sodium chloride, 5000 U of thrombin was added, resulting in a solution containing 1429 U thrombin per mL. The solution was aspirated with a 1 mL syringe to fill the PICC lumen.
4. Using a 5 mL sterile syringe, U3 mL of sterile anticoagulant bovine blood at 37°C was drawn. It was slowly injected into a clean and dry test tube along the tube wall. Subsequently, 0.1 mL of vitamin K1 was extracted using a 1 mL syringe and added to the test tube. The mixture of sterile anticoagulant bovine blood and vitamin K1 was gently rotated to blend.
5. The 1 mL empty syringe was connected to the infusion joint. The PICC tip connected to the joint was immersed in sterile bovine blood mixed with vitamin K1.
6. Sterile bovine blood (0.15 mL) mixed with vitamin K1 was aspirated, and the catheter was clamped at a distance of 5 mm from the PICC tip.

### 2.3 Model Preparation

The prepared model of thrombotic catheter occlusion was maintained in a vertically suspended position at room temperature, specifically 22°C–24°C, for 72 h.

## 3 Determination of Catheter Occlusion Model

### 3.1 Basis for Determination

Based on the Standards of Practice for Infusion Therapy (Gorski et al. 2021), the criteria for catheter occlusion include:

1. Absence of blood reflux or slow blood flow upon withdrawal of the syringe plunger.
2. Decreased drip rate during infusion; resistance when pushing the plunger or inability to flush the catheter lumen; inability to infuse fluids.
3. Repeated blockage alarms from the electronic infusion pump.
4. Swelling or leakage at the infusion site.
5. Absence or inadequate blood flow in the hemodialysis central venous vascular access device.

### 3.2 Operation Procedure

The model is removed and cut at the clip along the tip. The catheter tip of the model is placed in a container containing 0.9% sodium chloride, the infusion joint is removed, and the PICC is connected to a 10 mL syringe for aspiration. The catheter is withdrawn thrice, and blood reflux is recorded. Subsequently, the catheter is connected to the infusion apparatus and the infusion pump, and the occurrence of blockage alarms is recorded (with the blockage alarm pressure threshold of the infusion pump primarily set at 0.1–0.14 MPa). The thrombotic catheter occlusion model is considered to be successfully established if there is no blood reflux in three withdrawals of the syringe plunger and the infusion pump registers a blockage alarm.

## 4 Data Collection and Analysis

The data collected include:

1. Number of cases of catheter thrombosis modeling.
2. Number of cases of intra‐catheter thrombosis.
3. Number of cases of catheter occlusion determined by the syringe withdrawal method.
4. Number of cases of catheter occlusion determined by the infusion pump alarm method.
5. Number of cases of intra‐catheter thrombus overflow determined by the syringe withdrawal method and the length of the overflowed thrombus.
6. Number of cases of intra‐catheter thrombus overflow determined by the infusion pump alarm method and the length of the overflowed thrombus.

In this study, the chi‐square test was used to indicate the correlation between the results obtained from the syringe withdrawal method and the infusion pump alarm method, considering both as unordered categorical variables. Additionally, analysis of variance (ANOVA) was used to examine the differences in the length of overflowed intra‐catheter thrombus across various cases. Statistical analyses were conducted using IBM SPSS 25.0 software.

## 5 Results

### 5.1 Intra‐Catheter Thrombosis Model

A total of 140 models were produced, all of which exhibited thrombosis, resulting in a 100% success rate.

### 5.2 Thrombotic Catheter Occlusion Model

In this study, 118 models were successfully established, which were identified by withdrawal of the syringe plunger without blood reflux and triggering of the infusion pump alarm, resulting in a success rate of 84.29% (118 out of 140). There were 127 cases of withdrawal of the syringe plunger without blood reflux, constituting an incidence rate of 90.71%. In 13 cases, withdrawal of the syringe plunger resulted in blood reflux mixed with fine thrombosis, with an incidence rate of 9.29%; no thrombus overflowed from the catheter tip in these cases. There were 126 cases of infusion pump alarms, accounting for an incidence rate of 90%. In 14 cases where no infusion pump alarms were triggered, thrombus overflowed from the catheter tip, with an incidence rate of 10% (Table 1).

**TABLE 1:** Statistical analysis of the thrombotic catheter occlusion model.

Infusion pump	Cases	Syringe withdrawal		*χ* ^2^	*p*
		No blood reflux = 127	Blood reflux = 13		
Alarm	126	118	8	9.648^a^	0.002
No alarm	14	9	5		

^a^ The total number of cases ≥ 40, and the chi‐square test needs to be continuously corrected if there is a theoretical frequency ≥ 1 and < 5, and the result is subject to the continuous correction result.

Based on the chi‐square test, a statistically significant difference was found between the two assessment methods (*p* < 0.05), indicating a significant correlation between the results of syringe withdrawal and infusion pump alarms.

### 5.3 Intra‐Catheter Thrombus Overflow

There were nine cases where withdrawal of the syringe plunger resulted in no blood reflux and triggered an infusion pump alarm, all of which expelled a strip‐shaped thrombus. There were eight cases where withdrawal of the syringe plunger resulted in blood reflux and triggered an infusion pump alarm, revealing fine thrombosis in the blood reflux. The expelled thrombus was mostly finely fragmented. There were five cases where withdrawal of the syringe plunger resulted in blood reflux without triggering an infusion pump alarm. Fine thrombosis was observed in the blood reflux, and the expelled thrombus was a mixture of finely fragmented and strip‐shaped thrombus. Statistical results regarding the length of overflowing intra‐catheter thrombus are presented in Table 2 and between‐group test results are depicted in Table 3.

**TABLE 2:** Statistical results of the length of the overflowed intra‐catheter thrombus.

Groups	Cases	Mean ± standard deviation	*F*	*p*
1. Withdrawal of the syringe plunger without any blood reflux and no infusion pump alarm.	9	175.03 ± 41.826	11.519	0.001
2. Withdrawal of the syringe plunger resulting in blood reflux, accompanied by the absence of an infusion pump alarm.	5	85.45 ± 25.4700		
3. Withdrawal of the syringe plunger resulting in blood reflux, accompanied by activation of the infusion pump alarm.	8	131.31 ± 27.419		

**TABLE 3:** Statistical results of between‐group tests on the length of the overflowed intra‐catheter thrombus.

Groups	Groups	Mean difference	Standard error	Significance	95% confidence interval	
					Lower limit	Upper limit
The syringe plunger was withdrawn without any blood reflux or an infusion pump alarm.	2	89.5884	18.9163	< 0.001	49.9961	129.1808
	3	43.7232	16.4793	0.016	9.2317	78.2147
2 Withdrawal of the syringe plunger with blood reflux and no infusion pump alarm	1	−89.5884	18.9163	< 0.001	−129.1808	−49.9961
	3	−45.8653	19.3340	0.028	−86.3317	−5.3988
3 The syringe plunger was withdrawn due to blood reflux and an infusion pump alarm.	1	−43.7232	16.4793	0.016	−78.2147	−9.2317
	2	45.8653	19.3340	0.028	5.3988	86.3317

The homogeneity test of variance for the three groups of data yielded *p* > 0.05. Based on the variance test, the data among the three groups revealed *p* < 0.05, indicating a significant statistical difference in thrombus length among the groups. Between‐group tests revealed significant statistical differences among the three groups. In the group where withdrawal of the syringe plunger resulted in no blood reflux and no infusion pump alarm, the average thrombus length was 175.03 mm, the longest among the groups. In the group where withdrawal of the syringe plunger resulted in blood reflux without triggering an infusion pump alarm, the thrombus length averaged 85.45 mm, the shortest among the groups.

## 6 Discussion

### 6.1 Principles and Key Points of Thrombotic Catheter Occlusion Modeling

Wang identified three factors influencing thrombosis: blood stasis, hypercoagulability, and vascular endothelial damage, with the latter triggering significant release of coagulation factors (Wang et al. 2020). In this study, the intra‐catheter thrombotic occlusion did not encompass vascular endothelial damage. Instead, it focused on key thrombotic factors like blood viscosity, red blood cell density, thrombus morphology and distribution, and blood clotting time.

In a study conducted by Schwein, a large animal model of deep vein thrombosis in the iliac vein and vena cava was established by inducing venous blood flow arrest using a balloon catheter and inducing hypercoagulability through thrombin injection (Schwein et al. 2022). In this research, the catheter lumen was pre‐filled with thrombin solution prior to blood withdrawal to induce hypercoagulability, leading to the formation of intra‐catheter thrombus. To prevent blood leakage and maintain blood flow arrest, the distal opening of the catheter was clamped. The catheter drooping method was used to ensure the cross‐section of the catheter lumen was filled with intra‐catheter thrombus, with the catheter placed in a drooping position. Due to gravity and erythrocyte sedimentation principles, red blood cell concentration near the catheter tip increased, increasing blood viscosity and ensuring the morphology and distribution of the thrombus.

Hitchcock demonstrated that intraluminal blockage can occur unpredictably with the PICC tip positioned correctly, sometimes shortly after placement (Hitchcock 2016). In this study, the modeling duration was limited to 72 h. This was to ensure the firm adhesion of thrombus to the catheter wall and maintain the hypercoagulable state of blood within the catheter, as well as to enhance the concentration of red blood cells.

Based on the aforementioned principles and key control measures, a total of 140 catheter models were generated in this study, achieving a 100% success rate in intra‐catheter thrombosis production. Among these models, 118 cases successfully established thrombotic catheter occlusion models, resulting in a success rate of 84.28%.

### 6.2 Judgment Method of Thrombotic Catheter Occlusion

In studies conducted abroad on catheter occlusion, the definition of catheter occlusion is variable (Hermite et al. 2012; Parienti et al. 2014; Quenot et al. 2019; Souweine et al. 2015; Schallom et al. 2012; Pe'rez‐Granda et al. 2014). Based on the Standards of Practice for Infusion Therapy, catheter occlusion is defined as the obstruction of a vascular access device lumen that impedes the ability to flush and/or infuse solutions or withdraw blood through the lumen (Gorski et al. 2021). This study adopted the definition of catheter occlusion from the Standards of Practice for Infusion Therapy, along with the recommended methods and criteria for assessment (Gorski et al. 2021). The primary methods used to determine catheter occlusion include withdrawal of the syringe plunger and assessment of blood reflux, as well as flushing the pipeline.

Thrombotic catheter occlusion occurs when a blood clot completely fills the cross‐sectional area of the lumen due to intra‐catheter thrombosis, leading to blockage. In this study, catheter occlusion was confirmed by observing blood clots in the reflux when withdrawing the syringe plunger, which revealed blood reflux. Additionally, in five cases without infusion pump alarms, a significant amount of blood clots could be expelled from the catheter tips. In the 17 cases with infusion pump alarms, blood clots were also expelled from the catheter tips. This observation highlights that the presence of thrombus within the catheter does not necessarily result in blockage. Moreover, the process of determining catheter blockage may lead to a two‐way thrombus flow.

The study results indicated a correlation between withdrawal of the syringe plunger without blood reflux and the activation of the infusion pump alarm, both effective methods for determining catheter occlusion. However, a 6.35% risk of thrombus dislodgment (8 out of 126 cases) was discovered in this study when using the infusion pump alarm method to assess catheter blockage in the presence of intra‐catheter thrombus. Conversely, the risk of thrombus dislodgment was 100% (14 out of 14 cases) when the alarm was not triggered. This highlights a potential risk of thrombus dislodgment into the body when relying on the infusion pump alarm method, indicating a preference for using the syringe withdrawal method for assessment.

### 6.3 Clinical Significance of This Study

#### 6.3.1 The Impact of Intra‐Catheter Thrombus Overflow In Vitro on the Daily Maintenance of Clinical Catheters

In this study, it was observed that in five cases where intra‐catheter thrombus was present, the syringe withdrawal method indicated unobstructed catheters, while the infusion apparatus alarm method identified thrombus overflow at the catheter tip, with an average thrombus length of 85.45 mm. This finding indicates that while the syringe withdrawal method can assess catheter patency, it does not reliably indicate the presence of thrombus within the catheter.

In clinical practice, routine catheter maintenance is carried out in accordance with the recommendations in the Standards of Practice for Infusion Therapy (Gorski et al. 2021). During daily flushing procedures, the adoption of pulsatile flushing technology is recommended to prevent blood stasis within the catheter lumen and maintain a smooth inner wall that discourages thrombus formation. Notably, these standards do not specify whether the presence of thrombosis within the catheter should be confirmed prior to flushing. Ornowska et al. demonstrated that nurses inject fluid into vascular access devices to maintain lumen patency when continuous medication infusion is not required (Ornowska et al. 2023). Positive pressure sealing involves injecting a sealing solution into the catheter to prevent blood from clotting in the lumen in the absence of flow. However, there is a risk that small blood clots may dislodge into the body during this procedure. Therefore, it is essential to confirm catheter patency and check for intra‐catheter thrombosis before undertaking clinical catheter maintenance procedures. Only after confirming these conditions should pulsatile flushing and positive pressure sealing be conducted to minimize the risk of thrombus dislodgement from the catheter tip into the blood vessels.

#### 6.3.2 Clinical Significance of the Visible Thrombosis in Blood Reflux When Withdrawing the Syringe Plunger

The Manual of Intravenous Therapy advises slowly withdrawing the catheter initially during flushing to observe for blood reflux, which should have the same color and consistency as whole blood (Gorski 2018). However, the manual does not specify whether the withdrawn blood should be reintroduced into the catheter after this observation or if it needs to be examined for thrombus. In practical catheter maintenance procedures, when blood reflux is observed, it cannot be drawn into the infusion joint or syringe but only into the catheter itself. Subsequently, the withdrawn blood is promptly pushed back into the catheter immediately after blood reflux is observed.

In this study, 13 catheters were determined to be unobstructed by the syringe withdrawal method, and the withdrawn blood was mixed with fine thrombus when intra‐catheter thrombus was present. Subsequently, the infusion pump method was used for assessment, revealing that in five cases without alarms, the length of thrombus overflowing from the catheter tip was shorter, whereas in eight cases with alarms, the length of thrombus overflowing was moderate. This finding indicates that even if the catheter is deemed unobstructed by the syringe withdrawal method, the presence of thrombus in the blood reflux indicates a risk of dislodgement during fluid infusion, potentially leading to thrombus migration into the bloodstream. Catheter occlusion is confirmed when an alarm is initiated during pressure infusion using the infusion pump. However, before the infusion pump alarm is activated, more thrombus may flow into the bloodstream, potentially becoming migratory thrombus, which can cause pulmonary embolism and other serious conditions. Even if there is no alarm, a small amount of thrombus might still enter the bloodstream. Therefore, when using the syringe withdrawal method to determine catheter occlusion, it is essential to check the withdrawn fluid for thrombus. If a thrombus is present, it should be aspirated from the catheter. Infusion should only be continued after manual pulsatile flushing to ensure that the thrombus is removed.

#### 6.3.3 Clinical Significance of Intra‐Catheter Thrombus Pumped Out of the Catheter Tip by the Infusion Pump

The Standard of Practice for Infusion Therapy indicates that frequent pump alarms are among the criteria for determining catheter occlusion (Gorski et al. 2021). However, it does not explicitly mention that using an infusion pump may lead to a bolus injection of a blood clot into the patient. Hitchcock noted that when a vascular access specialist assesses a PICC for occlusion, the primary intervention involves flushing the catheter lumen using the pulsatile flushing technique (Hitchcock 2016). If resistance is encountered during flushing, a common approach to restore patency involves sealing the tube with 10 U/mL of heparin saline to restore patency.

In this study, there were nine cases of withdrawal of the syringe plunger with no blood reflux and no infusion pump alarm, all of which exhibited overflowed thrombus, with an average length of 175.03 mm, accounting for 64.29% of the infusion pump non‐alarm models. This indicates a high risk of thrombus overflow from the catheter in such cases, with the length of migratory thrombus flowing into the blood vessel being longer. Therefore, when assessing PICC occlusion, the pulsatile flushing technique should not be used, nor should the infusion pump alarm be relied upon for judgment. Otherwise, the risk of thrombus overflow from the catheter is significantly increased.

#### 6.3.4 Provision of Model for Clinical Study of Catheter Occlusion Recanalization Methods

The thrombotic catheter occlusion model can be used to compare the efficacy of various catheter recanalization methods. This model is key in identifying catheter dysfunction at an early stage, enabling preemptive intervention.

### 6.4 Limitations of Modeling

This model is an in vitro experimental model and, therefore, cannot accurately simulate the in vivo environment, including factors like intravital blood flow dynamics and blood viscosity. In this study, only a thrombotic catheter occlusion model was developed. However, actual clinical catheter occlusion is more complex and may involve drug‐induced catheter occlusion, thrombotic catheter occlusion, and mixed catheter occlusion.

## 7 Conclusion

In this study, the modeling method of thrombotic catheter occlusion was reliable. The model effectively simulates the basic characteristics of thrombotic catheter occlusion, indicating potential application in clinical practice. It is recommended that the pulsatile flushing technique not be used for catheters with suspected thrombotic occlusion. Additionally, the infusion pump alarm method is not advised for judging catheter occlusion. When using the syringe withdrawal method to determine catheter occlusion, it is essential to check for thrombus in the withdrawn fluid. If a thrombus is present, fluid infusion is not recommended.

## Author Contributions

Conception and design of the research: Feng‐Xian Li, Qiao‐hong Guo. Acquisition of data: Feng‐Xian Li. Analysis and interpretation of the data: Feng‐Xian Li, Qiao‐hong Guo. Statistical analysis: Feng‐Xian Li. Obtaining financing: None. Writing of the manuscript: Feng‐Xian Li. Critical revision of the manuscript for intellectual content: Qiao‐hong Guo. All authors read and approved the final draft.

## Ethics Statement

The authors have nothing to report.

## Consent

The authors have nothing to report.

## Conflicts of Interest

The authors declare no conflicts of interest.

## Data Availability

The datasets used and/or analyzed during the current study are available from the corresponding author upon reasonable request.
